# Development and Evaluation of a Multimodal Supportive Intervention for Promoting Physical Function in Older Patients with Cancer

**DOI:** 10.3390/cancers14112599

**Published:** 2022-05-24

**Authors:** Eni Shehu, Sigrid Roggendorf, André Golla, Antonia Koenig, Gabriele I. Stangl, Andrea Diestelhorst, Daniel Medenwald, Dirk Vordermark, Anke Steckelberg, Heike Schmidt

**Affiliations:** 1 Institute of Health and Nursing Science, Medical Faculty, Martin Luther University Halle-Wittenberg, 06112 Halle, Germany; shehu.eni@gmail.com (E.S.); sigrid.roggendorf@uk-halle.de (S.R.); anke.steckelberg@uk-halle.de (A.S.); 2 Institute of Rehabilitation Medicine, Medical Faculty, Martin Luther University Halle-Wittenberg, 06112 Halle, Germany; andre.golla@uk-halle.de; 3 Institute of Agricultural and Nutrition Science, Martin Luther University Halle-Wittenberg, 06120 Halle, Germany; antonia.koenig1@gmail.com (A.K.); gabriele.stangl@landw.uni-halle.de (G.I.S.); 4 Clinic and Polyclinic of Radiotherapy, University Hospital Halle, 06120 Halle, Germany; andrea.diestelhorst@uk-halle.de (A.D.); daniel.medenwald@uk-halle.de (D.M.); dirk.vordermark@uk-halle.de (D.V.)

**Keywords:** cancer care, older patients with cancer, physical activity, physical function, nutrition, health-related quality of life

## Abstract

**Simple Summary:**

Physical function is important for older people to maintain selfcare and independence. Physical function may decline during oncologic therapy. In this study, a program to help maintain physical function was developed and tested. Cancer patients, 60 years and older, starting outpatient radiotherapy participated. The individual health condition, risk factors and quality of life were assessed. The results informed individual exercise plans and dietary recommendations. Participants received either paper-based or video-based instructions. After 12 weeks of intervention the assessments were repeated. Four weeks later, a questionnaire was sent to ask about physical activity, nutrition and quality of life. Twenty-four patients participated (14 women, 10 men) with a mean age of 70 ± 7 years. The majority rated the program as helpful. Paper-based or video-based instructions were appreciated equally. The intervention was feasible and showed potential benefit for the maintenance of physical function during outpatient radiotherapy and should be tested with a larger sample.

**Abstract:**

Physical function (PF) in older patients with cancer may decline during and after oncologic therapy. This study aimed to develop and pilot test an individually tailored unsupervised physical activity (PA) program and dietary recommendations to promote PF in older patients with cancer. Following development and pretest, the intervention was pilot tested to explore feasibility, acceptance, adherence and potential benefit. Patients ≥60 years, with heterogeneous cancer diagnoses, starting outpatient radiotherapy were randomized in two study arms: paper-based vs. video-based instructions. Based on assessments of PF, PA, nutrition, cognition, mental health, social support, HRQOL and personal goals, participants received individual recommendations for PA and nutrition. After 12 weeks of intervention (T1), reassessments were performed. The postal 4-week follow-up questionnaire included PA, nutrition and HRQOL. Participants (*n* = 24, 14 female, mean age 70 ± 7 years) showed comparable characteristics in both study arms. The majority rated the program as helpful. Facilitators and barriers to PA adherence were collected. Both modes of instructions were appreciated equally. PF (EORTC QLQ-C30) declined slightly (not clinically relevant >10 pts.) at group level T0: 76 ± 16, T1: 68 ± 21, T2: 69 ± 24. The intervention was feasible, well accepted, showing potential benefit for the maintenance of PF during outpatient radiotherapy, and should be further tested in a larger sample.

## 1. Introduction

Older people with cancer are heterogeneous regarding their biological age, functional capacity and the number and severity of comorbidities. International guidelines recommend a comprehensive assessment of physical, psychological and cognitive function, nutritional status and social support in older people with cancer prior to therapy [1]. However, despite tailored therapies and effective treatments, physical function (PF), mobility, activities of daily living (ADL) and health-related quality of life (HRQOL) may be impaired in the long term and to a critical degree [2,3]. 

Derks et al. described that younger breast cancer patients were able to regain their PF status after therapy, while older (70+) patients showed a lower functional level one and two years after therapy [4]. However, in women with breast cancer, Hurria et al. also described resilience, i.e., recovery from a decline during therapy in 47% while one-third still experienced a decline at 12 months [5]. For very old patients (≥80 years) with heterogeneous diagnoses undergoing radiotherapy, a small observational study reported reduced PF six months after discharge [6]. Another study combining the assessment of risk factors prior to therapy with transsectoral care also reported clinically relevant reduced PF and role function in combination with increased fatigue at the 6-month follow-up [7]. 

Numerous studies show positive effects of physical activity (PA) in adults with cancer [8,9,10] and research interest regarding physical activity (PA) for older adults with cancer is increasing [11,12,13]. To enhance positive effects, guidelines recommend dietary counselling complementary to exercise training [14]. Studies showed positive effects of PA interventions for older patients with cancer including dietary recommendations regarding HRQOL and PF [15,16]. The combination of PA and healthy eating may also improve therapy tolerance and HRQOL [17] and may prevent sarcopenia [18]. However, the number of studies examining PA interventions for older patients with cancer is still limited when compared with the evidence for adults with cancer [11,19,20]. Furthermore, access to oncological training therapies is often limited for older patients with cancer in rural areas [19]. Therefore, this study was designed to develop and pilot test a home-based intervention comprising an individual PA program and dietary recommendations for older patients with cancer undergoing outpatient radiotherapy. The primary aim of this study was to evaluate the feasibility of the entire multimodal intervention. Secondary aims were to explore feasibility and acceptance of different modes of access with digital (DI) versus paper-based instructions (PI) and the potential benefit of the intervention.

## 2. Materials and Methods

Development and pilot testing were conducted according to the UK Medical Research Council (MRC) framework for the development and evaluation of complex interventions [20]. The reporting follows the Criteria for Reporting the Development and Evaluation of Complex Interventions in healthcare: revised guideline (CReDECI 2) [21].

### 2.1. Study Design

The study design comprised a stepwise approach with development, pretest and pilot test. For the pilot test, aiming to examine the feasibility of the intervention in a heterogeneous population, the setting of outpatient radiotherapy was chosen as radiotherapy (RT) which is applied to a large percentage of cancer patients with heterogeneous diagnoses and is also well-tolerated by older patients. As this study was a pilot study, we intended to cover a wide variety of standard treatment concepts with therapy plans ranging from high dosage short treatments, e.g., 70.4 Grays (Gy) to lowest dose of 50 Gy applied for a maximum of 32 fractions, so that participants with differing risk for side effects could be included. 

The intervention was intended to start with the beginning of RT with an intervention time of 12 weeks. Depending on the therapy concepts, the duration of active RT varied. Therefore, RT was not applied during the whole intervention time.

While the content of the intervention was comparable for all participants, the pilot testing was designed as a non-blinded randomized parallel trial with two intervention arms for digital instructions (DI) and paper-based instructions (PI), to explore feasibility and acceptance of the different modes of access and presentation of the instructions (secondary aim). The website www.randomizer.org (accessed on 10 May 2022) was used for the generation of random assignments for binding 1:1 allocation of participants to the groups. Blinding of the participants was not feasible as the DI group received tablet computers.

### 2.2. Inclusion Criteria

Inclusion criteria were age 60 years or older, confirmed diagnosis of cancer and beginning of outpatient radiotherapy treatment. Exclusion criteria were severe cognitive impairment (e.g., dementia), acute mental illness (e.g., psychosis), insufficient knowledge of the German language precluding an understanding of the instructions or the completion of the questionnaires, medical contraindications for PA (e.g., bone metastases at risk of fracture, fever, thrombocytopenia), life expectancy shorter than 6 months or diagnosis of severe cardiac insufficiency (NYHA—stage IV).

### 2.3. Program Development and Pretest 

**Figure 1:**
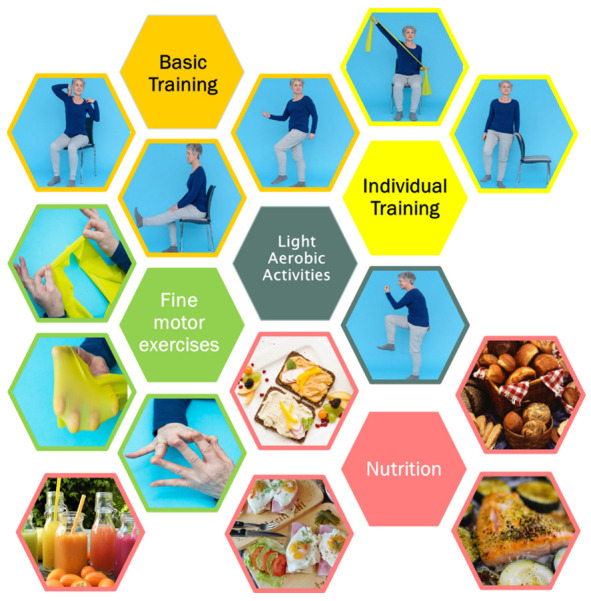
Exemplary elements of the physical activity program and dietary recommendations.

The methodological approach was based on the transtheoretical model considering individual needs, barriers and facilitators and the integration of training routines into everyday life [22]. Based on existing evidence [12,23,24], a portfolio with exercises which were considered safe and easily adaptable for different difficulty levels including sitting and standing positions was compiled. Aiming to maintain and practice capabilities and motions relevant for everyday functioning, exercises for endurance, strength, coordination and fine motor skills were included in the portfolio. Instructions comprised explanations of all single movements and exercises either with photos (paper-based instructions, PI) or with videos (digital instructions, DI). Single exercises were combined to “join-in” motion sequences for “basic training” of the upper and lower body parts in sitting and standing position. The videos included an additional music option to increase motivation during the exercise sessions. The basic training focused on endurance, strength and coordination. In addition, to meet special needs, specific exercises for the training of strength without (*n* = 4) and with resistance rubber bands (*n* = 10), and one sequence for balance and coordination were included. Addressing the issue of finger strength and function which is relevant especially for older people, e.g., for grasping, opening of medication bottles, etc., targeted exercises for fine motor skills of fingers (*n* = 7) were also included (Figure 1). Further examples are given in the Supplementary Materials, Box S1 basic training and Box S2 examples of specific exercises. Based on international guidelines [14], nutritional recommendations comprised information about macronutrients (e.g., proteins, carbohydrates, fats), micronutrients (e.g., vitamins) and fibers. In addition, the dietary recommendations included information and self-management advice for symptoms and side effects of the oncologic therapy (loss of appetite or taste, difficulties eating, chewing or swallowing, oral mucositis, dry mouth, nausea and vomiting, bloating, obstipation, diarrhea, unwanted weight gain and unwanted weight loss). In addition, easy recipes were compiled for helping and motivating participants to cook and eat (Figure 1).

The pretest, which aimed to evaluate the applicability, acceptance and comprehensibility of the developed materials, was conducted with a convenience sample of older cancer survivors. In three pretest sessions, the practical exercises and instructions were reviewed [25]. Following optimization, all components (PA instructions, nutritional recommendations) and contraindications for PA were discussed and consented with experts from the fields of radiation oncology, rehabilitation medicine, cardiology, psychology, sports science, nursing science and nutrition.

### 2.4. Pilot Study 

Recruitment and all assessments for the pilot study took place in the Clinic and Polyclinic of Radiotherapy, University Clinic Halle Saale (UKH). If applicable, travel costs were reimbursed. Eligibility was determined by the treating physicians.

#### Procedure

The comprehensive baseline assessments comprised PF, HRQOL, social contacts and situation, emotional status, cognition, activities of daily living (ADL), physical activity (PA) and a 3-day nutrition diary. Depending on their previous knowledge, participants were informed about the benefits of PA during cancer treatment and individual PA goals were discussed. Based on this information, an individual PA plan was conceptualized. Frequency, intensity, timing and type of the recommended exercises were planned according to the results of the physical performance tests, aiming for moderate intensity PA six days/week. Recommendations included alternating activities with basic training and/or walking aiming for approximately 30 min./day according to the individual condition and the number and scope of the targeted specific exercises. Recommended repetitions and series, e.g., with resistance rubber bands, were documented in the PA plan. Resistance rubber bands of suitable intensity for strength exercises were provided. Minimum duration of the motion sequences (e.g., basic training and coordination) was predefined by the music in the video-based group (appr. 5 min./sequence). Repetitions were encouraged. Participants of the PI group were instructed to use a timer or clock to monitor duration. Examples of the PA plan are provided in the Supplementary Materials, Figure S1 PA plan.

The selected exercises were explained and first-time execution by the participants was supervised. If necessary, the PA plan was adjusted. Individual barriers and facilitators possibly influencing the implementation at home were discussed. Participants were given pedometers to record daily steps and a PA diary to document their activities on a daily basis, including wellbeing (smileys). They were also instructed how to use the Borg Scale to reflect and document the perceived intensity of the exercises (Borg Scale) in this diary (Figure S2 PA diary). Participants were encouraged to observe and appreciate their individual state of health and capability which may change during the day and depending on treatments and if necessary to adapt intensity and frequency either independently or in consultation with the first author (ES). One week later, another face-to-face meeting focused on conduction and feasibility of the exercises at home including perceived barriers and/or facilitators. Adaptions were made if necessary and participants continued to execute the exercises independently at home. For questions or problems, a call-in option was available for all participants every working day. Based on the nutrition diary and their nutritional status, participants received dietary recommendations. Duration of the whole intervention was planned for 12 weeks.

### 2.5. Outcomes and Methods of Data Collection 

Data collection included three measurement points: baseline (T0), end of the 12-week intervention (T1) and four-week follow-up (T2). Respective methods of data collection for each time-point are described below.

#### 2.5.1. Baseline Assessments (T0)

Patient-reported PF was assessed with the EORTC QLQ-C30 complemented by physical performance measures and self-reported PA.

Reference values for interpretation of physical performance were based on literature [23,24,26,27,28].

1. Physical performance measures: timed up and go test (TUG) [29] dual-task TUG [30], 6 min walk test (6 mWT) [31], hand-grip strength [32], five-times sit-to-stand test (5TSTS) [33] and 4-stage balance test [34].
2. Self-reported PA: Physical Activity Scale for the Elderly (PASE) [35] and sport biography [36] PASE Score. Usual range: 0–400; 7-day recall.
3. Nutrition: 3-day nutrition diary.
4. ADL [37] and Instrumental ADL (IADL) [38].
5. Social contacts and situation [39].
6. Cognition: mini-mental state examination (MMSE) [40] and clock drawing test [41].
7. Emotional status: Patient Health Questionnaire (PHQ-9) [42].
8. Patient-reported HRQOL: EORTC QLQ-C30 [43] and EORTC QLQ-EDL14 [44]. Scale range 0–100; recall period last week.

These assessments provided a comprehensive picture of the physical condition complemented by psychosocial aspects and cognitive function, thus informing communication and matching of the individual recommendations. For maximizing the participants’ benefits of this program, new findings from the study-related assessments were reported to the clinical staff and, if indicated, added to the clinical record.

#### 2.5.2. Post-Assessments (T1)

All assessments were repeated at the end of the intervention (T1).

Feasibility was assessed through recruitment and drop-out rate. In addition, a semi-structured interview was conducted to explore feasibility and participants’ experiences with the intervention and the study materials, acceptance, motivation, adherence to the PA program including facilitators, barriers and individual independent adjustments made by the participants, perceived benefits, suggestions for improvements, future plans for PA and diet. Adherence was also assessed via diaries and pedometers. In addition, any changes regarding nutrition between the counselling and the end of the intervention including barriers were explored.

#### 2.5.3. Follow-Up (T2)

The postal follow-up (T2) comprised the EORTC QLQ-C30 (the EORTC ELD-14 was not included to reduce burden of the participants), the PASE questionnaire and open-ended questions about the maintenance of PA after the intervention (“My current PA are…” and “My plans to continue being physically active are…”). Regarding nutrition, changes since the end of the intervention were explored (“The changes I made to my nutrition since our last meeting are…” and “My plans to continue eating well consciously are….”).

### 2.6. Data Analysis

Analysis of qualitative data was conducted according to Mayring comprising a combination of inductive and deductive procedures [45] with MAXQDA 2018 (VERBI software, Berlin, Germany).

For quantitative data descriptive analyses, mean values (MV) and standard deviations (SD) were calculated using the commercial software IBM SPSS Statistics 25.0. Descriptive analyses of HRQOL (EORTC QLQ-C30) also comprised symptom and functioning scales and spaghetti plots to illustrate individual trajectories of PF (EORTC QLQ-C30). Descriptive analysis of physical function over time were only performed for complete data sets of the EORTC QLQ-C30 for all three measurement times and in addition for all available data and both study arms. For HRQOL, a mean change of 10 points between two measurement times was considered “clinically relevant” [46]. Descriptive comparisons of physical performance measures were performed for participants with available data for T0 and T1 and in addition for all available data and both study arms. Potential benefits were explored through descriptive comparisons of physical performance and patient-reported PF, selected functioning and symptom scales and single items of the EORTC QLQ-C30 and EORTC QLQ-EDL14 between T0 and T1.

## 3. Results

### 3.1. Results of the Pretest

Eight cancer survivors (five female, aged between 60 and 80+) participated in the pretest. They reported various persisting PF limitations, e.g., decreased balance, coordination and strength, and barriers regarding the attendance of rehabilitation training groups due to the high difficulty level and/or limited access in rural areas. Participants reported challenges through everyday tasks such as dressing or housework, walking or biking and underlined that they tried to be physically active every day. They valued the concept of combining PA and nutritional recommendations, rated the nutritional information as very helpful and gave feedback regarding the presentation of the materials (e.g., larger font, less text, selection of music) [23].

### 3.2. Results of the Pilot Study

Recruitment took place from 8 June 2020 to 15 February 2021. Out of 35 eligible patients, 24 patients gave informed consent to participate and were randomized into two groups (DI *n* = 13; PI *n* = 11). Reasons for non-participation are listed under feasibility. There was one lost before follow-up (cause: death).

#### 3.2.1. Sample Description 

Demographic and clinical characteristics of all participants are presented in Table 1.

#### 3.2.2. Outcomes

In the following section, we will firstly describe the qualitative results of the semi-structured interviews regarding feasibility including acceptance and adherence. We also briefly report qualitative results regarding perceived benefit, facilitators and barriers for PA and nutrition, as these factors might possibly influence motivation, adherence and thus potential benefit.

We will then present the quantitative results regarding potential benefit including assessments of physical performance, nutrition and HRQOL. Separate results for both study arms (DI and PI) are presented in the Supplementary Materials.

##### Feasibility

Flow of participants: Eleven participants refused to participate in the study because of satisfaction with their individual PA level (*n* = 6), time concerns or daily obligations (*n* = 2) and other reasons not specified (*n* = 2).

At T1, 21 participants completed the assessments at the clinic. Due to the inability to travel to the clinic, two participants were interviewed by telephone.

The T2 follow-up questionnaire was returned by *n* = 19 participants.

Changes in the planned realization: Due to the required time, in some cases, the assessments at T0 were conducted at two meetings.

One participant was bound to a wheelchair and had limited use of the hands and could not perform respective assessments. Individual exercises for strength and shoulder mobility were recommended. At T0 and T1, two and four participants, respectively, refused the 6 min walk test due to time concerns and absence of a walking aid.

One participant stopped the PA program after 4 weeks reporting to be already active but was still interested to continue contacting the nutritional scientist (AK) and to follow the nutritional recommendations.

Three participants were nourished via a feeding tube (*n* = 3); therefore, the nutrition-related content was not applicable. Two participants declined nutritional counselling stating that the recommendations of dietitians in general were not implementable in everyday life and they did not want feedback about their eating habits due to former bad experiences with nutritional counselling at other centers.

Acceptance and perceived usability of study materials for PA: 

Regarding digital access, 11 out of 13 participants of the DI group reported that the tablet PCs were easy to use (e.g., regarding charging, starting and access to the exercise program and nutritional information), and the initial reported issues regarding charging (*n* = 1) and finding the exercise program (*n* = 1) were solved at the second face-to-face meeting.

The tablet computers were well accepted; however, some participants were reluctant at the beginning. *“I am always so afraid that I break something, and then it won’t work—but when you explained it again...This was very new for me, but afterwards it went great” (P10).*

The written information was rated as understandable, clearly explained, including appropriate and sufficient explanatory text. 

Use of pedometers varied between the participants and over time (Supplementary Materials, Figure S3). Seventeen participants used the pedometer (min. one week, max. 12 weeks). Some participants found the pedometer to be motivating (*n* = 6), whilst others found it bothersome (*n* = 3).

The PA diaries with daily recording appealed to some participants (*n* = 10 returning complete diaries) while others stopped completing the diaries, as they had no changes to report. Therefore, the available data do not represent the actual activities of the whole sample. 

Participants’ feedback on the PA program: The PA program was predominantly rated positively. Participants reported that the exercises were helpful during therapy, they enjoyed the exercises and that PA was fun or associated with wellbeing. *“During radiotherapy it was good, also the feeling that I “did it „gave me a sense of accomplishment” (P02).* After some time, according to their needs, some participants adjusted the PA program independently. *“At some point you start to do it longer yourself. You make an effort when you do it and after a certain time you start to do more. I got a yellow resistance band, I but I switched to a green one that I have at home”.* They valued the opportunity to integrate the basic program in everyday life, e.g., mobility exercises, when sitting or waiting somewhere. *“When I’m bored, I almost automatically do this [demonstrates the hand exercise]” (P09).* Two participants rated the exercises as not helpful for their special goals, e.g., related to a degenerative shoulder problem and radiotherapy-related fatigue.

Regarding suggestions for optimization of the PA program, some participants wished a wider range of lower body exercises. Participants in the digital group suggested additional handouts with the PA plan and short instructions, as they did not need the videos after a while, having mastered the exercises.

Reported factors possibly influencing adherence to the PA program:

Qualitative analysis revealed the following facilitators regarding adherence to the PA plan: social support (relatives or friends), environmental factors (good weather and accessibility of public parks), intrinsic motivation (wellbeing after exercises, not feeling well and bad conscience being inactive), regular exercising (e.g., specific time every day) and motivating elements of the program (e.g., physiotherapist, PA plan, pedometer or PA diary). *“You can stick to something like that [the exercise program], you have a task. We had a rhythm every morning” (P03)*.

Qualitative analysis also revealed barriers regarding adherence to the PA plan: health-related reasons (comorbidities, side-effects of cancer treatment), scheduling, lack of social support or living alone, weather and missing motivation. *At the beginning I did nothing or almost nothing, because I was not feeling well. It was only in the middle of January [after 6 weeks] that I started to do other exercises” (P22)*.

*“The exercises did not agree with the radiation appointments. There the whole day was lost with transportation, waiting and then, at home, I had to rest.”*

(P24)

*“I’m not the type to do it all by myself at home. The program works, but I would like someone to do it with me, to show me. When I meet someone that always motivates me, too.”*

(P12)

*“When the sun was shining I was fine, but when it is cloudy and there is no sun...I am not a person for it.”*

(P08)

*“Often I thought “not today”. One is not always motivated.”*

(P2)

Maintenance and change in activity behavior during follow-up:

Regarding maintenance and change in activity behavior at follow-up (T2), participants reported PA activities such as household chores (*n* = 14), walks (*n* = 14), leisure time sports, e.g., biking, gymnastics, swimming and rehabilitation classes (*n* = 11), Nordic walking (*n* = 9), shopping (*n* = 8), gardening (*n* = 7), continuation of the received PA plan (*n* = 6), child care (*n* = 3), medical appointments (*n* = 3) and animal care (*n* = 1). 

PA plans for the future were reported by *n* = 14 participants, e.g., gardening, walking in fresh air, biking, training groups, swimming, Nordic walking, endurance training and continuation of the PA program of this study. Participants expressed the desire to be more active but found that restrictions due to the COVID-19 pandemic or their current physical function level hindered them. *“I plan to be physically active, but: …I will do what my condition allows” (P14)*.

*“…I will attend fitness and group therapies when gym will open again.”*

(P16)

and *“…I still need to become painless and stronger.”*

(P18)

Feasibility and acceptance of study materials for nutrition: 

Regarding nutrition, feasibility and acceptance of the nutrition diaries varied. In total, 16 out of 24 participants completed the nutrition diaries at T0 and 9/16 at T1. Some participants found the diaries a burden, some delegated the task to spouses that were responsible for meal preparation. At T1, different reasons for non-completion were given such as problems with filling out the questionnaire.

Reported factors possibly influencing adherence to the dietary recommendations:

Adherence to dietary recommendations varied. Six participants adapted their nutrition after the counselling, e.g., eating more fatty fish, nuts, butter, double cheese, more vitamins and proteins, integrating some of the recipes in their daily diet or permitting themselves to eat what they liked and not exclude some ingredients from the diet. Participants mentioned that the awareness about eating habits also changed.

*“Less fat, with meat anyway, vegetables. […]. The awareness has changed. That’s good when you have these materials.”*

(P03)

Identified facilitators for implementing the nutritional recommendations in daily life included: determination (pushing yourself to eat), change in dietary patterns to reduce side effects (eating the dishes that were tasting good), nutritional support (e.g., in the rehabilitation clinic), rest, family support (e.g., partner), food supply (e.g., food stores, restaurants) and listening to the body (not forcing yourself to eat during mealtime, but only eating according to the desire to eat).

Participants valued reassurance regarding their nutritional habits, concrete information about optimization of their diets, self-management advice for symptoms, not being obliged to restrictions and the opportunity to discuss their uncertainties with the nutrition scientist. 

Identified barriers were attitudes (e.g., moral reasons for vegetarian diet), non-evidence-based recommendations from friends, family and media, cancer therapy or its side effects (e.g., nausea, loss of taste), family obligations (e.g., caring for relatives), depressive symptoms and not feeling up to coping with the situation.

Maintenance and change in eating habits during follow-up:

Regarding maintenance, during follow-up and future plans, *n* = 11 participants reported plans to eat healthy (e.g., more vegetables, fish, less meat) and find respective cooking recipes. Suggestions for improvements included, e.g., a clear guide on what to eat on certain days during and after therapy.

*“More recipes, maybe. Well, cooking training, if offered. […] questions about vitamins or minerals—my hair is coming back now, I would like to know what I should use.”*

(P12)

##### Assessments of Physical Performance

Results of the assessments of physical performance for those participants with available data for T0 and T1 are summarized in Table 2. Separate results for both study arms (DI and PI) are presented in the Supplementary Materials, Table S1.

##### Self-Reported Physical Activity PASE Score

Within the usual range for the score of 0–400, the physical activity level of the study participants was rather stable at both measurement times (PASE score *n* = 23, T0: 99 ± 45 T1: 92 ± 48), with a slight tendency towards decrease. This trend can be observed in the paper-based group (T0: 92 ± 32 T1: 86 ± 46) as well as in the digital group (T0: 104 ± 56 T1: 96 ± 51). The analysis of the individual activity dimensions shows a slight increase in walking and moderate sports (Results for both study groups are presented the Supplementary Materials, Table S2).

##### Nutrition

Evaluation of the nutrition diaries that were completed by 16/24 participants at baseline revealed that recommended energy and quantities of proteins and fibers were not achieved by the patients (Table 3). Data show that the reduced energy intake was mainly caused by the low consumption of carbohydrates. 

In total, 9 out of 16 participants completed the nutrition diaries again at the end of the intervention. Comparison of the available data showed that these participants consumed more fibers expressed as a percentage of the actual intake in relation to the recommended intake levels (T0: 60 ± 17 (28–88), T1 86 ± 27 (55–133), while consumption of other nutrients, including proteins, mainly remained unchanged.

##### Health-Related Quality of Life

Table 4 shows MV and SD for all functioning scales and symptom scales of the EORTC QLQ-C30 and EORTC ELD-14 over time. In the Supplementary Materials, in Table S3, MV and SD for these scales are presented in comparison for the baseline (*n* = 24) and for participants that completed the assessments at all three time points (*n* = 19), Figure S4 shows box and whisker plots for functioning scales over time and Figure S5 shows box and whisker plots for selected symptom scales over time. Separate results for both study arms (DI and PI) are presented in Table S4.

Comparison of the PF scale of over time (*n* = 19) showed no clinically relevant changes between T0, T1 and T2 neither for the whole group nor within the groups. A clinically relevant decline was reported for role function between baseline and T1, and for emotional function between T1 and T2. 

Regarding symptoms relevant for physical functioning, clinically relevant increases were reported for fatigue, pain and sleeplessness between the end of the intervention (T1) and follow-up (T2) (Table 4).

**Figure 2:**
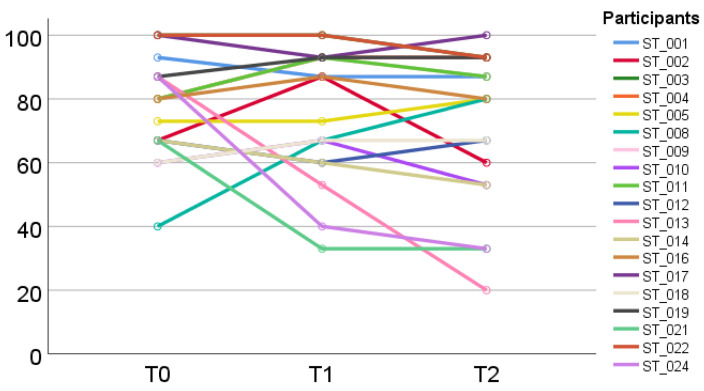
Individual development of physical function (EORTC QLQ-C30) from baseline (T0) to post-assessments (T1) and follow-up (T2).

Individual trajectories of patient-reported PF over time are shown (Figure 2) with *n* = 9 out of 19 participants reporting clinically relevant differences (≥10 Points).

## 4. Discussion

Results of this assessment-based intervention combining unsupervised PA and nutritional recommendations for older patients with cancer undergoing outpatient radiotherapy indicate feasibility and show potential benefits regarding the overall maintenance of patient-reported PF levels during intervention and follow-up. The qualitative results indicate comparable acceptance of the access modes and even a combination of digital and paper-based material for future studies. The descriptive quantitative results of assessments of physical performance for both groups indicate potential benefit for the maintenance though greatly influenced by the medical condition of the participants.

The results also provide relevant insights regarding perceived barriers and facilitators.

The relevance of such an intervention for this group is supported by Ursem et al. reporting declining PF during radiotherapy for patients without any intervention to support PF [47]. Similar results were found in a recent meta-analysis which concluded that exercise interventions are beneficial for PF in prostate cancer patients undergoing radiation therapy [48].

The intervention addresses research gaps identified in recent reviews regarding tailored nutritional advice and PA interventions, also considering the issue of behavioral change [15] and low-intensity interventions and intensified support for older people with physical limitations [49]. 

The chosen methodological approach according to the MRC framework [20] proved beneficial, as results of the pretest informed the specification of the intervention and results of the pilot testing provide relevant information regarding the feasibility and aspects to consider in future studies.

While feasibility, acceptance and adherence of the multimodal intervention itself was good, we firstly discussed issues that emerged and should be considered in future studies. These issues are also discussed in the limitations section.

The choice of assessments was based on recommendations for older patients with cancer [1] and specified to provide a holistic picture of physical, nutritional and psychosocial aspects possibly influencing PF and PA. The assessments were easily conducted, well accepted and informative for understanding participants’ resources and needs and tailoring advice for PA and nutrition. However, the integration of comprehensive assessments, timing and conduction in clinical routine proved a challenge especially for outpatients relying on support for transportation. 

Relevant findings from the study-related assessments were also used by the clinical team. The perceived clinical relevance of the assessments goes in line with results proving the clinical benefit of the CGA [50], predictive value of the CGA [51] and tests of PF [52,53,54]. However, despite this evidence and international recommendations, implementation of these assessments in oncological settings remains challenging and a systemic approach is needed to address organizational and economic barriers [55].

Regarding access, research examining the use of digital devices research is increasing, and these applications are the focus of studies for PA interventions in the older population [56,57]. As there are still prejudices regarding acceptance and use of digital media by older people, we aimed to examine acceptance in the target group separately with individual randomization to support a clear distinction and avoid contamination between the different modes of access. A clear distinction is also important regarding cost-benefit estimates for future studies, as both modes of access involve different resources for preparation and provision.

In our study, analyses showed no differences between the study arms with respect to feasibility, acceptance and adherence. The tablet-based PA instructions were well accepted, supporting the results of Mehra et al. who reported that the use of digital media for PA interventions was feasible for older aged individuals [56]. Regarding the use of digital media, our approach with face-to-face instructions to address individual insecurities goes in line with the conclusions of the qualitative study by Vaportzis et al. which argued that older adults are interested in the use of digital media and often only lack tailored instructions [58].

Participants valued the opportunity for home-based PA during the course of their outpatient radiotherapy treatment. They appreciated that individual limitations, goals, barriers and facilitators were discussed and taken into account for tailoring the exercise plan and nutritional recommendations. They valued the variety of exercises and the appropriate difficulty level leading to a sense of accomplishment. These results go in line with a systematic review assessing the PA needs and preferences of patients with cancer [59]. Participants underlined the role of experts to provide the program and a moderate difficulty level as facilitators for participating in PA, also reported by Wong et al. [59]. However, complementary to the review by Wong et al. which described that participants in most of the studies were interested in PA interventions after cancer treatment, our study showed that participants were also interested in a PA program during radiotherapy treatment.

All instructions were designed for unsupervised use in home settings aiming to increase confidence and autonomy for future maintenance. Motivational elements in our study comprised videos with instructions and “join-in” motion sequences with optional music and illustrations for the paper-based materials. Participants were encouraged to listen to their body signals aiming to empower them to adapt the exercises according to their condition independently.

The call-in option that was available during the intervention to support participants was not used often. Barrier-free means of communication especially for participants with speech impediments should be offered to provide an inclusive approach.

At the end of the intervention, achieved changes, individual barriers, facilitators and plans for the future were discussed. Regarding facilitators, the exercise plan and fixed schedules were perceived as supportive, which was also reported by Mikkelsen et al. [60]. Social and family support was found to increase motivation to exercise which is also described in other studies [60,61]. In contrast to other studies [60,62], participants in this study did not thematize anxiety as a barrier for PA; however, psychological support might have been appreciated in some cases.

Our results are in line with other studies examining unsupervised programs showing improvements in compliance in older adults [63] and benefits for patients that cannot easily access the specific training therapies e.g., due to living in rural areas [64]. Studies also showed cost effectiveness of unsupervised programs [65].

Our training program was designed to maintain everyday functioning. The PA level and PF for the whole group could be maintained while large interindividual differences in PA and PF depending on the individual medical condition and side effects such as fatigue were observed. While one participant explicitly reported that the exercises did not help to improve the experienced fatigue, MV for fatigue did not appear clinically relevant during the intervention for the whole group and both study arms. However, during follow-up, increases in MV for fatigue, pain and sleeplessness were reported. To address this decline, strategies for the maintenance of activity despite of symptom burden and high fatigue levels should be included in the intervention. Aiming to address the problem of fatigue, studies show the efficacy of targeted training [66]. Best results are reported for Yoga, strength and endurance [66,67]. To achieve lasting improvements in fatigue, moderate to vigorous intensity exercise with individualized prescriptions regarding frequency, intensity, time and type (FITT) are recommended [68]. In addition to fatigue, interventions addressing PF should include other patient-reported outcomes e.g., HRQOL and symptoms to tailor recommendations accordingly. In cross-sectional studies, Chen et al. reported inverse correlations of symptom clusters (e.g., pain, fatigue, sleep and nutrition related symptoms) with physical function [69] and Pandya et al. reported associations between high symptom burden with limitations of physical function, IADL impairments and falls [70]. Loh et al., in a recent review focusing on functional changes in older adults undergoing systemic cancer treatments, reported associations between functional decline and progressive disease status, pain, anemia and worse nutritional status [71].

Complementary to group-based MV and SD, especially in small samples such as our study, individual trajectories of PF (e.g., spaghetti plots) may complement quantitative data and allow a more case-based approach revealing individual conditions including possible associations e.g., between PF, symptom burden and the disease trajectory. Results of studies [69,70,71] and our findings highlight the relevance of repeated assessments of patient-reported symptoms to optimize supportive therapy during after care. As functional decline may impact HRQOL and selfcare, maintenance of PF constitutes a task for the multiprofessional team [72].

Regarding nutrition, the assessment-based nutritional information and recommendations were perceived as helpful. Corresponding to Hardcastle et al. [73], describing the need for easily accessible and individually tailored counselling, especially participants’ subjective needs due to anxiety and a lack of reliable information became evident and should be addressed. Regarding the implementation of nutritional advice, the distribution of domestic roles should be explored, as especially male participants often were not responsible for meal preparation and cancer may lead to changes in role function as described by Locher et al. [74].

### 4.1. Strengths and Limitations

Strengths of our study are the stepwise approach including pretest and expert consensus, to ensure suitability for the target group, the combination of PA and dietary recommendations based on patient-reported and objective assessments and the broad range and adaptability of exercises to address individual training needs. The intervention was tested in the context of clinical routine care revealing possible facilitators and barriers for the intervention itself and the patients using the intervention in their home settings. However, as this study was a pilot study, the results are not generalizable.

While the combination of different methods to monitor the physical activity behavior of the study participants during the intervention can be regarded as a strength, limitations of the used methods became evident.

The study has the following limitations: Assessment and interpretation of participants’ activities is limited due to missing pedometer and diary data. However, pedometers cannot capture upper body movements, gardening, swimming or biking [75]. Furthermore, the interpretation of the physical activity level (PASE score) is limited as it only refers to the last seven days before the two measurement time points. The PASE score is only a temporary activity measure and does not reflect the overall activity level during the intervention. Data of PA diaries may be biased due to social desirability [76], individual inaccuracies or unwillingness to record every activity [77,78]. In addition, nutrition related data are limited due to the low number of nutrition diaries.

While the number of recruited participants followed the recommendations for conducting pilot studies [79], the sample size of 24 participants is rather small regarding the heterogeneity of older patients with cancer. A possible selection bias regarding sedentary patients cannot be excluded as patients that were interested in improving their PA behavior participated. However, we consider this a low risk for bias as the sample comprised both active and inactive participants and one reported reason for non-participation was already being active [49]. Another possible selection bias might result from some patients’ declining participation because of time concerns. Time concerns were also reported due to transportation issues. In this case we offered the participants the opportunity to divide the first appointment into two or three appointments. This option might have influenced comparability.

With respect to assessments, the PHQ-9 comprises relevant diagnostic criteria for depression, some questions are possibly associated with the side effects of the cancer treatment (e.g., loss of appetite, problems with eating, concentration, remembering); therefore, the interpretation of PHQ-9 scores has to be made with caution.

### 4.2. Implications for Future Research

Results of this study regarding feasibility, acceptance and adherence can help design future studies. Regarding methodology, the duration of the assessments could be minimized by collecting patient-reported information regarding HRQOL, PA behavior and nutrition in advance via telephone or digital devices. Digital devices could also provide a more reliable method for monitoring PA [57]. Disabilities should be considered to provide inclusive alternatives and access to supportive options e.g., regarding speech impediments and call-in options. To further facilitate usability, a combination of printed and digital instructions would be advisable.

The results can inform the design of future studies with larger samples also including systemic cancer therapies and different age subgroups. Follow-up periods of at least 6 months could provide insights into long-term adherence and behavioral change as well as clinical benefits. In addition, future research should also examine the role of social relations which might be supportive or a hinderance to PA and nutrition. Regarding psychological aspects, personality traits, e.g., support seeking and the need for psychological support for patients and carers or significant others, should be taken into account.

## 5. Conclusions

Results indicate that the intervention is feasible and well accepted showing potential benefits regarding the maintenance of PF in older cancer patients undergoing outpatient radiotherapy despite individual variance. Further research is needed to verify these results.

## Figures and Tables

**Table 1:** Demographic and clinical characteristics of all participants (all), video-based group (DI) and paper-based group (PI).

	All (*n* = 24)	DI (*n* = 13)	PI (*n* = 11)
Age Mean ± SD (Min. Max.)	70 ± 7 (60–88)	70 ± 7 (63–88)	69 ± 7 (60–80)
Women *n* (%)	14 (58)	6 (46)	8 (73)
Education			
8 years	11	5	6
10 years	7	6	1
12 years	6	2	4
Vocational Training	11	7	4
Technical school	5	3	2
University	8	3	5
Cancer Site (*n* = 24)			
Head and Neck	8	6	2
Breast	7	3	4
Lung	5	2	3
Prostate	2	2	-
Gynecological	1	-	1
Brain	1	-	1
Tumor Classification (*n* = 22)			
T1	7	4	3
T2	9	5	4
T3	3	2	1
T4	3	1	2
N0	8	4	4
N1	9	5	4
N2	2	1	1
N3	2	1	1
M0	20	10	10
M1	1	1	-
Radiotherapy *	24	13	11
Surgery	15	8	7
Systemic therapy (e.g., Chemotherapy, endocrine/hormonal)	18	10	8
Comorbidities Mean ± SD (Range)	3 ± 2.4 (0–9)	3 ± 2.4 (0–8)	3 ± 3 (0–9)
Medications Mean ± SD (Range)	3 ± 3 (0–9)	4 ± 3 (0–9)	3 ± 2 (0–8)

* The applied RT concepts consisted mainly of normo- or mildly hypofractionated RTs. The maximum single dose was 2.66 Gy for breast cancer and the minimum 1.7 for head and neck cancer. Eleven patients received normofractionated RT.

**Table 2:** Objective assessments of physical performance of participants with available data for baseline (T0) and post-assessments (T1) categorized according to reference values.

Objective Assessments of Physical Performance	T0		T1	
	Reference Met	Reference Not Met	Reference Met	Reference Not Met
hand grip (*n* = 20)	19	1	18	2
TUG (*n* = 19)	19	0	19	0
dual task TUG (*n* = 14)	10	4	14	0
FTSTS (*n* = 19)	14	5	11	8
6 mWT (*n* = 13)	12	1	13	0
4-stage balance (*n* = 20)				
both feet	20	0	20	0
semi-tandem	20	0	20	0
tandem	19	1	17	3
one foot	18	2	16	4

**Table 3:** BMI (*n* = 24) and percentage of the actual in relation to the recommended energy and nutrient intake according to Arends et al. [14] of participants who completed the nutrition assessment (*n* = 16) (MV ± SD (Min. Max)).

Item	MV ± SD (Min. Max.)
BMI (kg/m^2^)	27 ± 2.8 (22.4–33.4)
Nutrient intake (% of the recommendation)	
Energy	73 ± 14 (47–105)
Protein	71 ± 15 (41–97)
Carbohydrates	63 ±12 (38–90)
Fat	88 ± 24 (46–142)
Fiber	60 ± 17 (28–88)

**Table 4:** Mean values (MV) and standard deviations (SD) for all functioning scales and symptom scales of the EORTC QLQ-C30 and EORTC ELD-14 over time.

EORTC QLQ-C30	T0 (*n* = 24) Mean ± SD	T1 (*n* = 23) Mean ± SD	T2 (*n* = 19) Mean ± SD
Physical Function	79 ± 16	75 ± 20	72 ± 24
Role Function	81 ± 29	70 ± 28	62 ± 33 *
Cognitive Function	85 ± 19	84 ± 17	79 ± 23
Emotional Function	69 ± 17	74 ± 20	56 ± 27
Social Function	79 ± 29	72 ± 31	74 ± 34
Fatigue	39 ± 25	42 ± 29	55 ± 27
Pain	30 ± 32	31 ± 37	50 ± 33
Nausea	9 ± 15	3 ± 8	6 ± 11
Dyspnea	39 ± 36	35 ± 34	37 ± 44 **^#^**
Sleeplessness	37 ± 33	43 ± 36	63 ± 35
Loss of Appetite	24 ± 36	24 ± 36 **^§^**	23 ± 33
Constipation	21 ± 32	16 ± 32	19 ± 34
Diarrhea	17 ± 31	8 ± 18 **^§^**	9 ± 19 *
Financial Problems	12 ± 22	10 ± 19	7 ± 18
Global Health Status	68 ± 19	63 ± 20	57 ± 23
EORTC ELD-14	**T0 (*n* = 24)** Mean ± SD	**T1 (*n* = 23)** Mean ± SD	
Mobility	28 ± 27	29 ± 30	
Joint Stiffness	32 ± 30	29 ± 32	
Worries about Others	58 ± 30	48 ± 28	
Future Worries	60 ± 27	49 ± 28	
Illness Burden	60 ± 25	56 ± 30	
Family Support	71 ± 37	64 ± 38	
Maintaining Purpose	80 ± 20	70 ± 26	

**^§^** T1 *n* = 22, * T2 *n* = 18, ^**#**^ T2 *n* = 17.

## Data Availability

There are no publicly archived data sets generated during the study. Data sharing was not included in the informed consent form and is therefore not possible. The authors can be contacted regarding the exercise program and dietary recommendations.

## Supplementary Materials

The following supporting information can be downloaded at: https://www.mdpi.com/article/10.3390/cancers14112599/s1, Supplementary Materials Box S1 basic training, Box S2 examples of specific exercises, Figure S1: PA plan; Figure S2: PA diary; Figure S3: Use of pedometers; Figure S4: EORTC QLQ-C30 Functioning Scales; Figure S5: EORTC QLQ-C30 Selected Symptom Scales. Table S1: Assessments of physical performance, activities of daily living, cognition, depression and social situation at baseline (T0) and after the intervention (T1); Table S2: PASE Scores; Table S3: Mean values (MV) and standard deviations (SD) for all functioning scales and symptom scales of the EORTC QLQ-C30 and EORTC ELD-14 over time; Table S4: Mean values (MV) and standard deviations (SD) for all functioning scales and symptom scales of the EORTC QLQ-C30 and EORTC ELD-14 for both study arms over time.
