# Supplementary material for: Development and Evaluation of a Multimodal Supportive Intervention for Promoting Physical Function in Older Patients with Cancer
Source: Cancers (Basel). 2022 May 24;14(11):2599. doi: 10.3390/cancers14112599 (PMC9179325; doi:10.3390/cancers14112599)

Sitting position

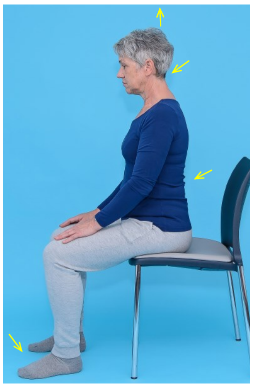

Posture

Standing position

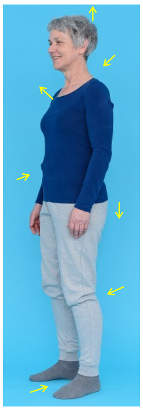

Exercises

heel raises

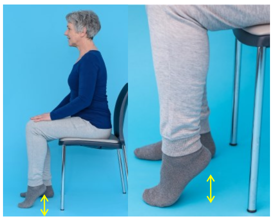

toe raises

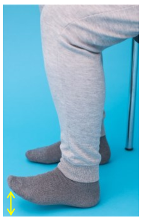

alternate heel/ toe raises

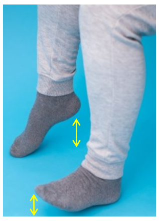

heel raises

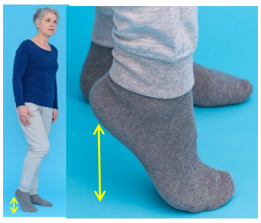

stand on tiptoes

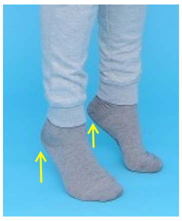

alternate heel raises

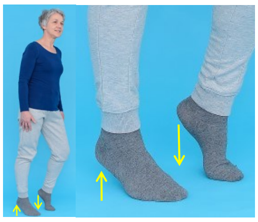

walking in place without and with reciprocal arm swing

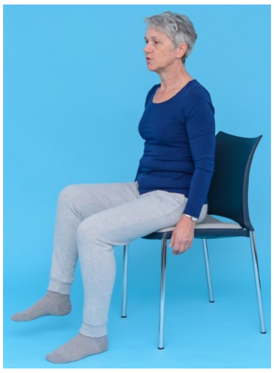

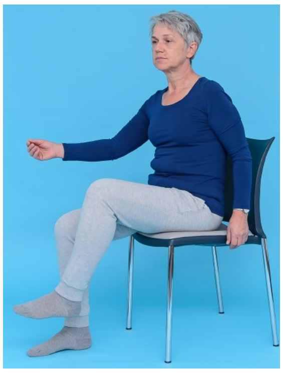

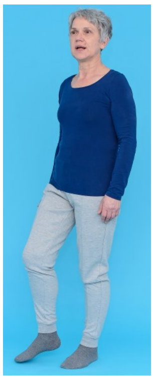

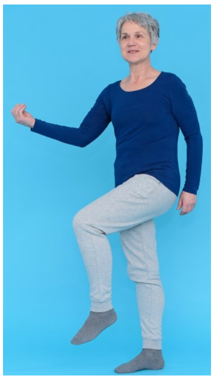

walking in place with reciprocal arm swing and slow turning of the head

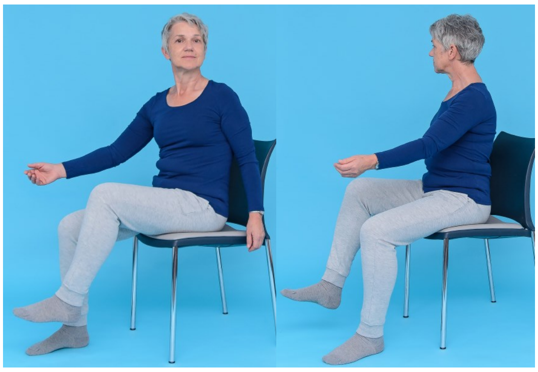

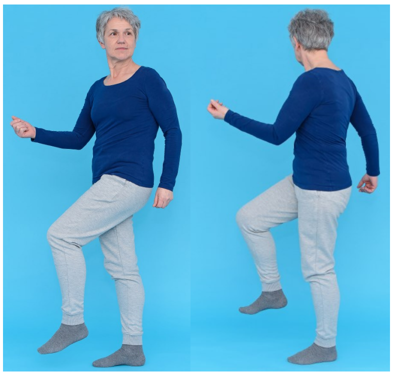

shoulder circles

forward

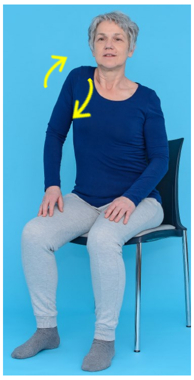

backward

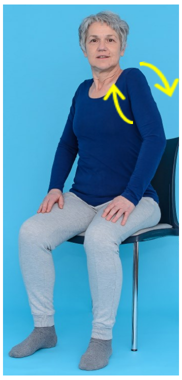

forward

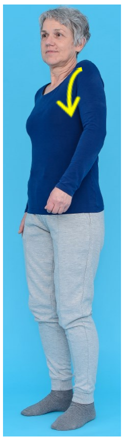

backward

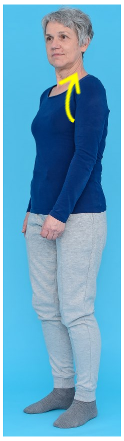

arm raises

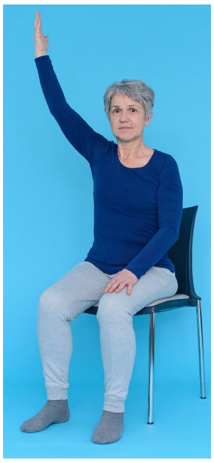

picking apples

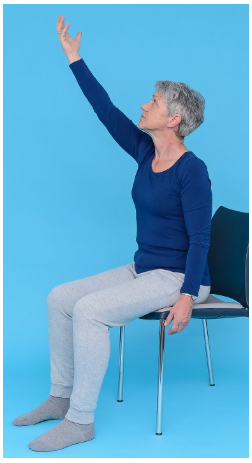

arm raises

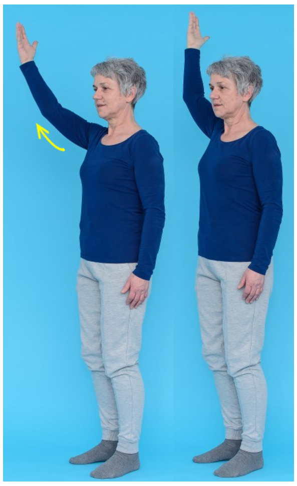

picking apples

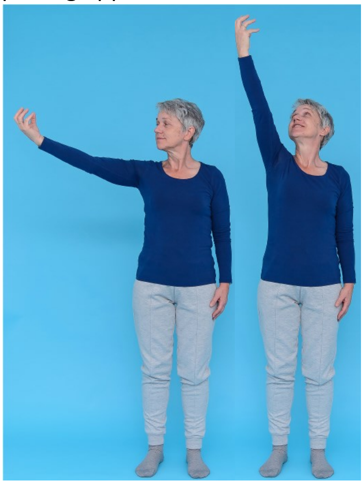

shoulder circles by rotating the elbow

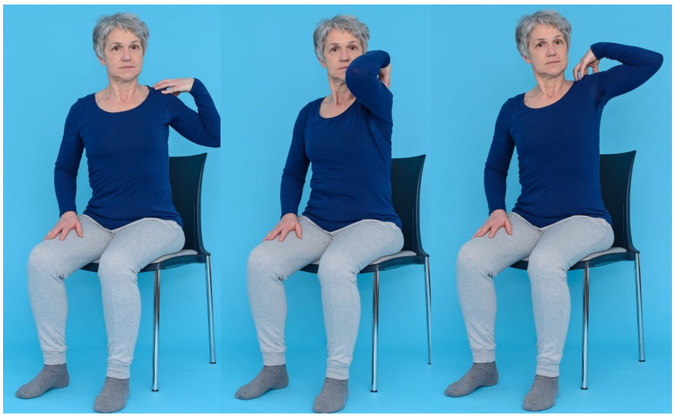

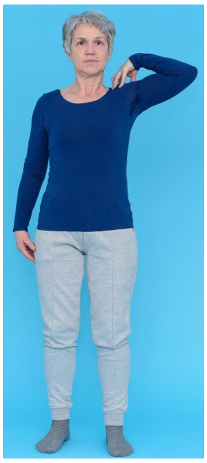

shoulder circles with both arms and trunk movement

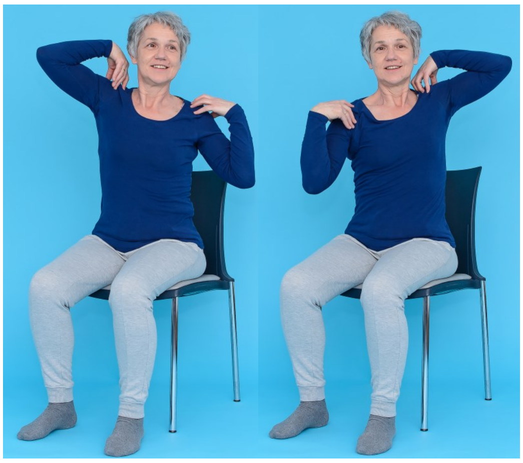

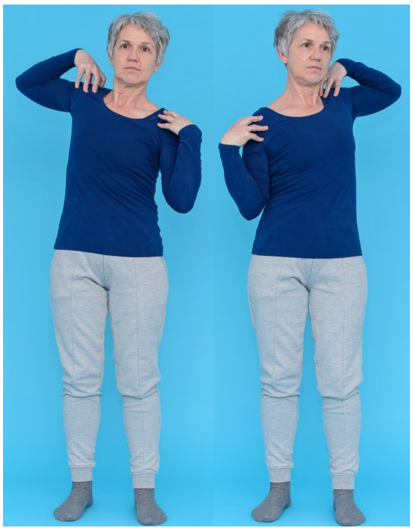

painting circles in the sky

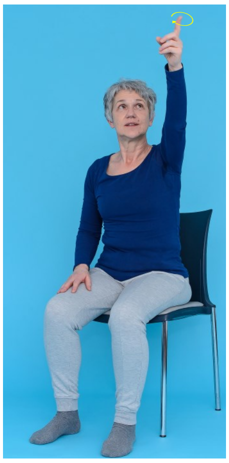

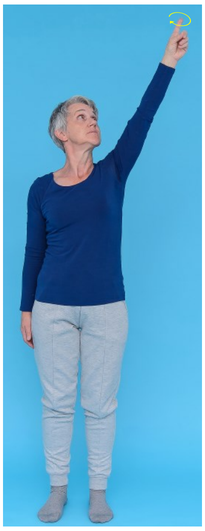

Supplement: Supplementary file 1 [file cancers-14-02599-s001.zip › supplementary material Box S1 basic training.pdf]
