# Supplementary material for: Development and Evaluation of a Multimodal Supportive Intervention for Promoting Physical Function in Older Patients with Cancer
Source: Cancers (Basel). 2022 May 24;14(11):2599. doi: 10.3390/cancers14112599 (PMC9179325; doi:10.3390/cancers14112599)
Supplement: Supplementary file 1 [file cancers-14-02599-s001.zip › supplementary material Box S2 examples of specific exercises.pdf]

Examples of specific exercises

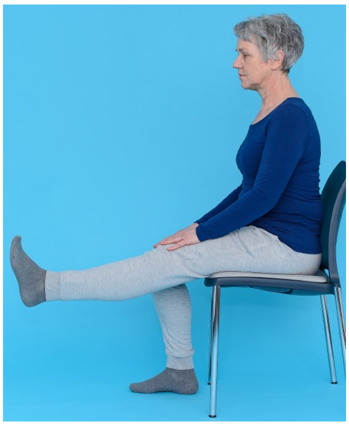

Knee extensions for strengthening legs in sitting position:

- pull up toes and straighten the knee as far as possible
- hold for some seconds (can be extended for enhancement)
- slowly set foot back down

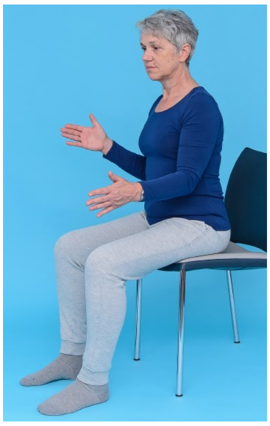

„Chopper“ for trunk strengthening and stability in sitting position:

- quick reciprocal movements of the forearms like chopping
- upper arms are fixed laterally at the trunk

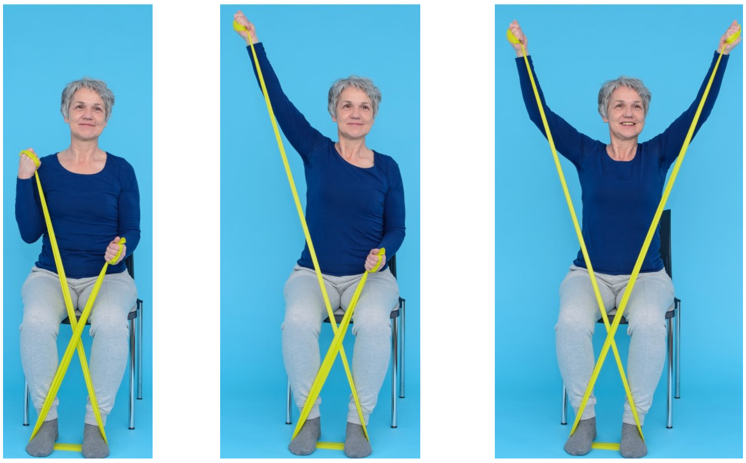

Exercises with rubber bands for strengthening arms and shoulders

- different ranges of arm movement address different muscles and levels of difficulty
- can be performed in a sitting or standing position
- also enhance trunk stability

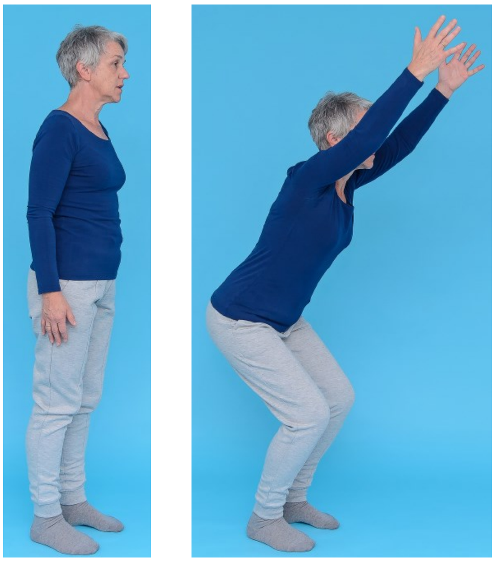

Knee bends for leg and trunk strengthening as well as improvement of balance

- bending knees
- lifting arms simultaneously

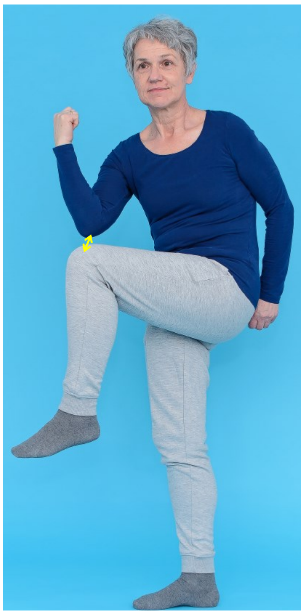

„Elbow meets knee“ for strengthening oblique abdominals and hip flexors and improving mobility

- can be performed in a sitting or standing position
- also improve balance when performed in the standing position

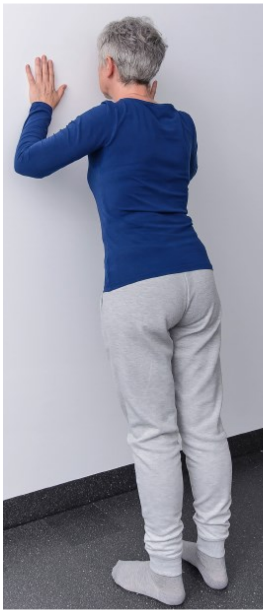

Press-ups at the wall

- Increasing standing distance or single-arm press-ups for enhancement

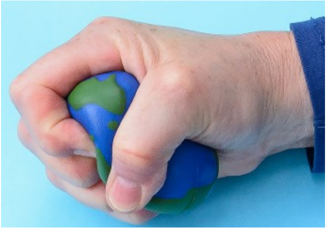

Squeezing a soft ball for grip strengthening:

- squeeze and hold for some seconds

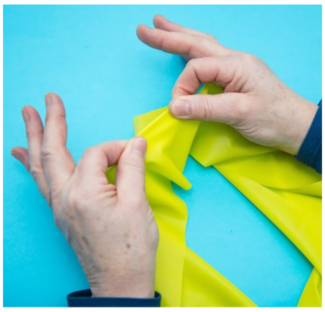

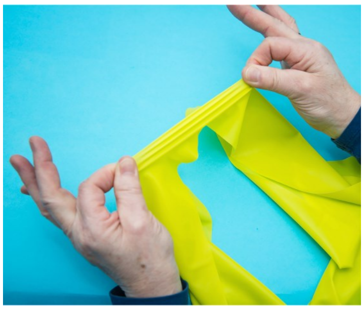

Stretching rubber band using pinch grip for strengthening and finger mobility:

- grasp with thumb and forefinger and stretch the rubber band
- repeating the same by grasping with thumb and each other finger
