# Supplementary material for: Development and Evaluation of a Multimodal Supportive Intervention for Promoting Physical Function in Older Patients with Cancer
Source: Cancers (Basel). 2022 May 24;14(11):2599. doi: 10.3390/cancers14112599 (PMC9179325; doi:10.3390/cancers14112599)
Supplement: Supplementary file 1 [file cancers-14-02599-s001.zip › supplementary material Graphics.pdf]

Activity plan for__[*NAME of the Participant]*__

Week: ______________

Figure S1. PA Plan.

	Day 1	Day 2	Day 3	Day 4	Day 5	Day 6	Day 7
Basic training lower body part	✓	✓	✓	✓	✓	✓	
Basic training upper body part	✓	✓	✓	✓	✓	✓	
Specific exercises upper body part	-Exercises with rubber band<br>-Press-ups<br><br>(3x10 reps/each)		-Exercises with rubber band<br>-Press-ups<br><br>(3x10 reps/each)		-Exercises with rubber band<br>-Press-ups<br><br>(3x10 reps/each)		*My reward?*
Specific exercises lower body part		-Knee extension<br>-Elbow knee<br><br>(3x10 reps/each)		-Knee extension<br>-Elbow knee<br><br>(3x10 reps/each)		-Knee extension<br>-Elbow knee<br><br>(3x10 reps/each)	
Walking/Biking	✓	✓	✓	✓	✓	✓	✓

This is an example of a physical activity plan for a patient that shows to be independent in everyday activities, has good balance skills and showed a good aerobic capacity in the baseline assessments (measured by six-minute walk Test) .

Figure S2. PA diary.

**Day**	**My activities**<br><br>**(Basic training, specific exercises, walking ….)**	**Perceived intensity**						**🕘 min.**	**After the exercises I feel …**	
		**low**		**moderate**		**high**			**physical**	**mental**
Monday									☺<br>😐<br>☹	☺<br>😐<br>☹
	Comments									

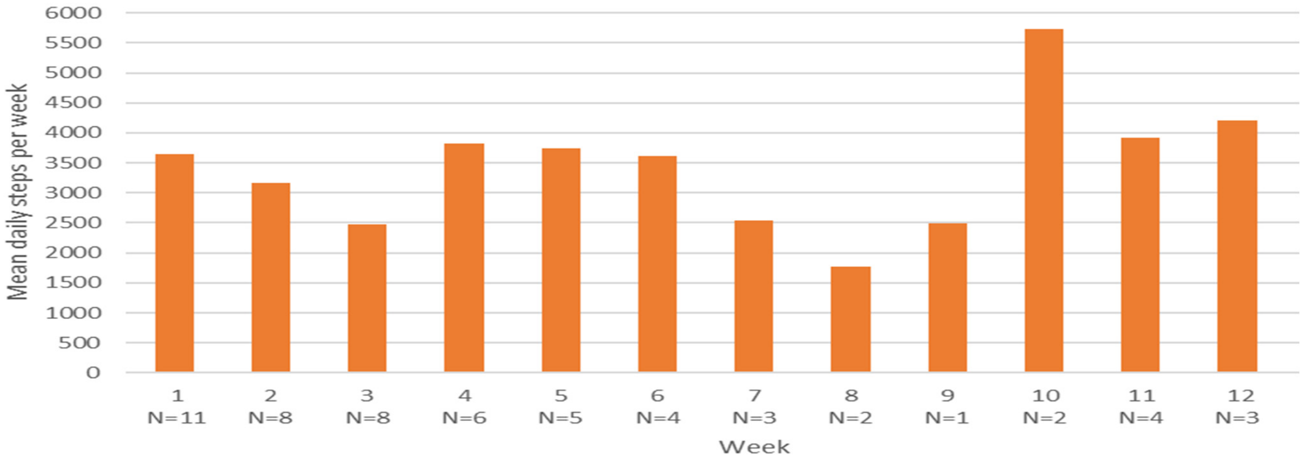

Figure S3. Mean daily steps per week of participants which used the pedometers more than 4 days for each given week.

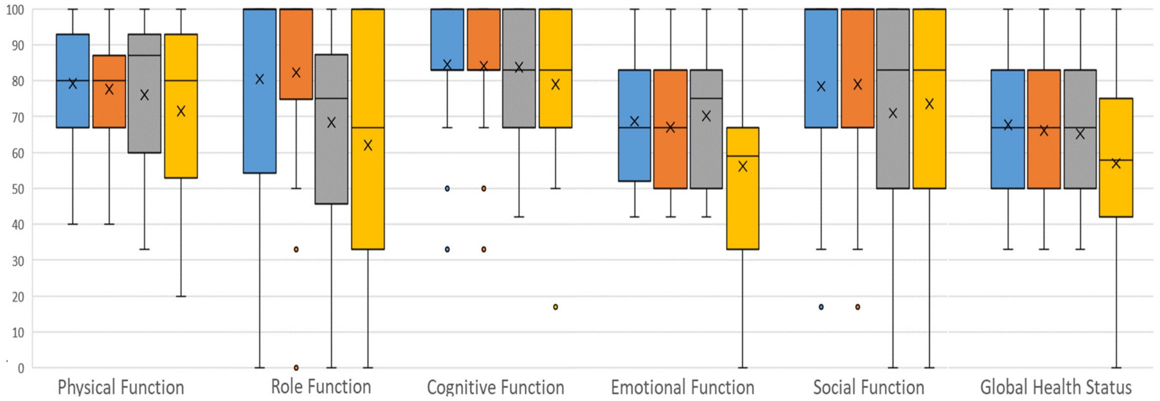

Figure S4. EORTC QLQ-C30 Functioning Scales (0-100 the higher the better) for baseline (n=24) (T0), baseline (n=19) (T0), post-assessments (n=19) (T1) and follow-up (n=19) (T2) *depicted with boxplots (box 50%, whiskers 25% quartiles, Median=line, Mean =x).*

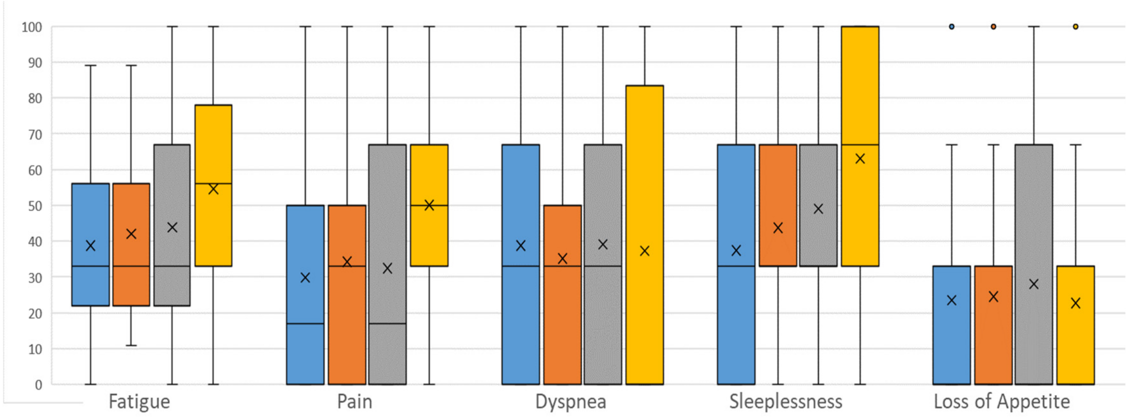

Figure S5. Selected Symptom Scales (EORTC QLQ-C30) (0-100 the lower the better) at baseline (n=24) (T0), baseline (n=19) (T0), post-assessments (n=19) (T1) and follow-up (n=19) (T2) *depicted with boxplots (box 50%, whiskers 25% quartiles, Median=line, Mean =x).*
