# Supplementary material for: Development and Evaluation of a Multimodal Supportive Intervention for Promoting Physical Function in Older Patients with Cancer
Source: Cancers (Basel). 2022 May 24;14(11):2599. doi: 10.3390/cancers14112599 (PMC9179325; doi:10.3390/cancers14112599)
Supplement: Supplementary file 1 [file cancers-14-02599-s001.zip › supplementary material Tables_May 24.2022.pdf]

Table S1: Assessments of physical performance, activities of daily living, cognition, depression, social situation and physical activity at baseline (T0) and after the intervention (T1) for both study arms DI and PI

		DI T0		PI T0*		DI T1		PI T1*#	
**Parameter**	Instrument (Scale)	N	MV ±SD (Min-Max)	N	MV ±SD (Min-Max)	N	MV ±SD (Min-Max)	N	MV ±SD (Min-Max)
**Physical performance**	Hand grip (kg) right	13	30±8 (16-41)	10	26±8 (17-39)	12	30±9 (17-43)	8	25±7 (18-36)
	Hand grip (kg) left	13	27±8 (17-41)	10	26±5 (20-34)	12	29±9 (18-45)	8	25±5 (20-36)
	TUG (sek)	13	8±1 (7-11)	10	8±1 (6-11)	12	7±2 (5-11)	7	7±1 (6-8)
	TUG-dual (sec)	11	10±2 (7-13)	8	10±2 (7-13)	11	9±2 (5-11)	6	9±1 (7-11)
	FTSTS (sec)	13	11±3 (6-16)	10	14±3 (10-17)	12	11±2 (8-15)	8	13±3 (10-21)
	6mWT (m)	11	470±90 (300-640)	10	397±101 (270-561)	8	499±85 (400-640)	6	487±69 (390-560)
	**4-Balance Test (sec.)**		10 sec aim reached		10 sec aim reached		10 sec aim reached		10 sec aim reached
	Both feet	13	13	10	10	12	12	9	8
	Semi-tandem	13	13	10	10	12	12	9	8
	Tandem	13	12	10	10	12	9	9	8
	One foot	13	10	10	9	12	9	9	7
		N	MV ±SD (Min-Max)	N	MV ±SD (Min-Max)	N	MV ±SD (Min-Max)	N	MV ±SD (Min-Max)
**Activities of Daily living**	ADL Items (0-4)	13	4±0 (4-4)	11	4±1 (2-4)	13	4±0 (3-4)	10	4±1 (1-4)
	IADL Items (9-0)	13	9±0 (8-9)	11	7±3 (2-9)	13	8±2 (0-9)	10	7±3 (1-9)
**Cognition**	MMSE	13	28±2 (25-30)	11	28±3 (19-30)	11	29±1 (27-30)	9	29±1 (26-30)
	Clock drawing test	12	2±1 (1-3)	9	2±1 (1-3)	11	2±1 (1-4)	8	1±1 (1-3)
**Social Situation**	Nikolaus	13	5±1 (3-6)	11	6±1 (4-6)	13	5±1 (2-6)	10	6±1 (4-6)
**Emotional Status**	PHQ-9	13	4±4 (1-14)	11	5±3 (0-9)	13	6±5 (0-16)	10	7±3 (1-12)

*T0 and T1: one participant was bound to a wheelchair and had limited use of hands

#T1: due to inability to travel to the clinic, two participants were interviewed by telephone

Table S2: PASE-Score results (range of the Scale 0-100; recall period 7 days) for the whole sample and for both study arms

		**All (nT0=24; nT1=23)** M±SD (min-max)	**DI (n=13)** M±SD (min-max)	**PI (nT0=11; nT1=10)** M±SD (min-max)
Walking at T0		18.6±17.3 (0-85.7)	18.5±10.1 (0-25.6)	18.8±23.7 (2.1±85.7)
Walking at T1		24.0±15.0 (5-51.4)	24.0±17.1 (5-51.4)	24.0±12.6 (6.4-51.4)
Light activities at T0		5.9 ±6.4 (0-27.0)	5.5±5.3 (0-15.8)	6.3±7.8 (0-27.0)
Light activities at T1		5.7±4.5 (0-15.8)	7.2±4.8 (0-15.8)	3.8±3.3 (0-9.0)
Moderate activities at T0		3.3±7.1 (0-29.6)	4.2±8.5 (0-29.6)	2.2±5.1 (0-14.8)
Moderate activities at T1		5.4±12.3 (0-59.1)	8.5±15.8 (0-59.1)	1.3±2.8 (0-7.4)
Vigorous activities at T0		2.5±12.1 (0-59.1)	4.5±16.4 (0-59.1)	0
Vigorous activities at T1		1.4±6.2 (0-29.6)	2.5±8.2 (0-29.6)	0
Special Muscle Training at T0		0.9±3.2 (0-12.9)	1.7±4.3 (0-12.9)	0
Special Muscle Training at T1		0.6±2.9 (0-12.9)	1.0±3.6 (0-12.9)	0
PASE Score at T0		94.8±49.6 (4.39-228)	104.3±56.3 (42.6-228)	83.6±39.9 (4.4-139.7)
PASE Score at T1		91.7±48.3 (8.8-183.9)	95.9±51.3 (10.3-183.9)	86.2±46.2 (8.6-145.4)
PASE Score per age groups				
60-64 years old	PASE Score at T0 (n=5; nID=2; nIP=3)	78.8±42.4 (36.7-134.2)	88.4±64.8 (42.6-134.2)	72.5±36.7 (36.7-110.0)
	PASE Score at T1 (n=5; nID=2; nIP=3)	107.6±44.0 (42.3-165.8)	134.8±43.8 (103.9-165.8)	89.4±40.9 (42.3-116.0)
65-69 years old	PASE Score at T0 (n=10; nID=6; nIP=4)	118.3±51.8 (54.9-228)	120.5±66.9 (54.9-228.0)	114.9±23.6 (85.7-139.7)
	PASE Score at T1 (n=10; nID=6; nIP=4)	90.6±53.6 (8.6-183.9)	88.1±51.6 (45.2-183.9)	94.4±64.4 (8.57-145.4)
70-74 years old	PASE Score at T0 (n=3; nID=2; nIP=1)	59.4±11.9 (50.7-73.0)	61.8±15.7 (50.7-73.0)	54.4
	PASE Score at T1 (n=3; nID=2; nIP=1)	87.2±66.4 (31.4-160.6)	115.1±64.3 (69.6-160.6)	31.4
75-79 years old	PASE Score at T0 (n=4; nID=2; nIP=2)	93.7±36.4 (50.7-139.7)	95.2±62.9 (50.7-139.7)	92.1±2.7 (90.3-94.0)
	PASE Score at T1 (n=4; nID=2; nIP=2)	98.1±16.4 (86.5-121.3)	103.9±24.6 (86.5-121.3)	92.4±7.9 (86.8-98.0)
80+ years old	PASE Score at T0 (n=2; nID=1; nIP=1)	73.1±97.1 (4.39-141.7)	141.7	4.39
	PASE Score at T1 (n=1; nID=1; nIP=0)	10.3	10.25	0

Table S3: Mean values (MV) and Standard Deviations (SD) for all functioning scales and symptom scales of the EORTC QLQ-C30 and EORTC ELD-14 for the whole sample and for participants with available data for all three measurement points (n=19).

**EORTC QLQ-C30**	**T0 (n=24) Mean ±SD**	**T0 (n=19) Mean ±SD**	**T1 (n=19) Mean ±SD**	**T2 (n=19) Mean ±SD**
Physical Function	79±16	78±16	76±20	72±24
Role Function	81±29	82±30	68±28	62±33*
Cognitive Function	85±19	84±19	84±18	79±23
Emotional Function	69±17	67±17	70±19	56±27
Social Function	79±29	79±30	71±32	74±34
Fatigue	39±25	42±24	44±30	55±27
Pain	30±32	34±32	33±39	50±33
Nausea	9±15	10±16	3±8	6±11
Dyspnoea	39±36	40±38	37±35	37±44#
Sleeplessness	37±33	44±34	49±36	63±35
Loss of Appetite	24±36	25±38	28±37	23±33
Constipation	21±32	21±34	14±32	19±34
Diarrhoea	17±31	21±34	9±19§	9±19§
Financial Problems	12±22	14±23	10±19	7±18
Global Health Status	68±19	66±19	65±21	57±23
EORTC ELD-14	T0 (n=24) Mean ±SD	T0 (n=19) Mean ±SD	T1 (n=19) Mean ±SD	
Mobility	28±27	30±28	32±31	
Joint Stiffness	32±30	33±31	33±33	
Worries about others	58±30	63±27	51±29	
Future Worries	60±27	63±29	54±26	
Illness Burden	60±25	64±24	61±29	
Family Support	71±37	74±36	70±33	
Maintaining Purpose	80±20	80±20	73±24	

* T2 n=18, § T1, T2 n=18, # T2 n=17

Table S4: Mean values (MV) and Standard Deviations (SD) for all functioning scales and symptom scales of the EORTC OLQ-C30 and EORTC ELD-14 at all measurement time points for both study arms DI and PI

	T0		T1		T2	
**EORTC QLQ-C30**	**DI (n=13)** Mean ±SD	**PI (n=11)** Mean ±SD	**DI (n=13)** Mean ±SD	**PI (n=10)** Mean ±SD	**DI (n=10)** Mean ±SD	**PI (n=9)** Mean ±SD
Physical Function	83±13 (60-100)	75±19 (40-100)	76±18 (40-100)	73±24 (33-100)	68±26 (20-93)	76±21 (33-100)
Role Function	88±22 (33-100)	71±34 (0-100)	70±31 (0-100)	68±25 (33-100)	57±36 (0-100)	69±29 # (33-100)
Cognitive Function	87±19 (33-100)	82±19 (50-100)	79±18 (42-100)	83±17 (50-100)	77±25 (17-100)	82±23 (50-100)
Emotional Function	72±15 (50-100)	64±19 (42-100)	85±18 (42-100)	67±21 (42-100)	55±25 (0-83)	57±31 (0-100)
Social Function	81±28 (17-100)	76±31 (17-100)	69±37 (0-100)	77±23 (32-100)	72±37 (0-100)	76±33 (0-100)
Fatigue	34±22 (0-67)	44±28 (11-89)	37±26 (11-100)	47±32 (0-100)	57±27 (22-100)	52±29 (0-83)
Pain	26±26 (0-67)	35±39 (0-100)	18±32 (0-100)	49±38 (0-100)	45±31 (0-100)	56±36 (0-100)
Nausea	9±13 (0-33)	9±17 (0-50)	1±5 (0-17)	5±11 (0-33)	3±7 (0-17)	9±15 (0-33)
Dyspnoea	36±35 (0-100)	42±40 (0-100)	33±33 (0-100)	37±37 (0-100)	52±44 # (0-100)	21±40 (0-100)
Sleeplessness	44±34 (0-100)	30±31 (0-100)	46±37 (0-100)	40±35 (0-100)	63±37 (0-100)	63±35 (0-100)
Loss of Appetite	18±32 (0-100)	30±41 (0-100)	21±35 (0-100)	30±39 # (0-100)	27±41 (0-100)	18±24 (0-67)
Constipation	13±22 (0-67)	30±41 (0-100)	5±12 (0-35)	30±43 (0-100)	17±32 (0-100)	22±37 (0-100)
Diarrhoea	18±32 (0-100)	15±31 (0-100)	8±20 (0-67)	7±15 # (0-33)	10±16 (0-33)	8±24 (0-67)
Financial Problems	10±16 (0-33	15±27 (0-67)	5±12 (0-33)	17±24 (0-67)	7±14 (0-33)	7±22 (0-67)
Global Health Status	70±15 (50-92)	65±24 (33-100)	65±15 (42-83)	60±26 (33-100)	53±25 (0-75)	61±21 (33-100)

	T0		T1	
EORTC ELD-14	**DI (n=13)** Mean ±SD	**PI (n=11)** Mean ±SD	**DI (n=13)** Mean ±SD	**PI (n=10)** Mean ±SD
Mobility	20±19 (0-56)	37±33 (0-100)	26±29 (0-78)	34±32 (0-78)
Joint Stiffness	28±18 (0-67)	36±41 (0-100)	18±22 (0-67)	43±39 (0-100)
Worries about others	50±32 (0-100)	68±25 (33-100)	44±21 (11-83)	54±35 (0-100)
Future Worries	60±30 (0-100)	61±26 (22-100)	40±29 (11-100)	61±22 (22-100)
Illness Burden	60±25 (17-100)	61±26 (17-100)	24±34 (0-100)	58±24 (17-100)
Family Support	64±44 (0-100)	79±27 (33-100)	54±42 (0-100)	77±28 (33-100)
Maintaining Purpose	83±17 (50-100)	76±23 (33-100)	74±26 (17-100)	65±25 (33-100)

one participant did not complete this scale
